# Histopathological Findings of Testicular Tissue Following Cadmium Toxicity in Rats

**DOI:** 10.30699/IJP.20201.130581.2443

**Published:** 2021-07-06

**Authors:** Saeedeh Shojaeepour, Shahriar Dabiri, Bahram Dabiri, Masoud Imani, Maryam Fekri Soofi Abadi, Fatemeh Hashemi

**Affiliations:** 1 *Pathology and Stem Cell Research Center, Kerman University of Medical Sciences, Kerman, Iran*; 2 *Department of Pathology, NYU Winthrop, Mineola NY, USA*; 3 *Department of Clinical Sciences, Faculty of Veterinary Medicine, Shahid Bahonar University of Kerman, Kerman, Iran*

**Keywords:** Cadmium toxicity, Rat, Testis, Sertoli cell

## Abstract

**Background & Objective:**

Reproductive toxicity of cadmium (Cd) as an environmental toxicant has been proved in animals and humans. Exposure to Cd impairs testes organs and can reduce male fertility. The present study was designed to investigate the spectrum of histopathological changes in testicular tissue focusing on Sertoli cells in rats following Cd intoxication.

**Methods:**

In the present experiment, acute testicular toxicity was induced by an intraperitoneal injection of 1.2 mg/kg CdCl_2_ to the animals in the test group, while the control group received normal saline. After 52 days, the animals were euthanized, and testicular tissue was stained by Hematoxylin and Eosin. In addition, immunohistochemical staining was performed on Sertoli cells for Wilms' Tumor, Melan-A, and CD99 to evaluate histopathological changes.

**Results:**

Cd caused significant alterations in seminiferous tubules with varying effects on the patterns of spermatozoa production. These histopathological changes were significantly higher in the Cd group, compared to the control group.

**Conclusion:**

The Cd-induced stepwise spectrum changes included sloughing, disorganization, hypospermatogenesis, spermatic cell arrest, germ cell hypoplasia, Sertoli cell-only pattern, fibro-hyalinized seminiferous tubules, and calcification. Sertoli cells accumulated and created multinucleated giant cells in the seminiferous tubules during the atrophic process, which could be dependent upon Sertoli cells viability and function.

## Introduction

Approximately 15% of couples experience infertility which affects their life. Almost 50% of these cases are attributed to male factors, such as impaired spermatogenesis. Environment and lifestyle are impor-tant factors influencing men's fertility. Therefore, male reproductive health is certainly sensitive to environ-mental exposures and pollution (1). 

Cadmium (Cd) is one of the most well-known environmental pollutants causing severe health problems. This element is distributed in the environment, including soil leading to the contamination of plants and the food chain. People with diets based on crops, such as rice and soybean are more exposed to Cd toxicity. One of the major sources of Cd absorption is tobacco smoking as a cigarette contains 5 mg of this metal leading to extraordinary blood Cd levels of 4–5 times higher in smokers, compared to non-smokers (2).

Cd poisoning is a global concern and can be associated with diverse diseases, namely cancers, diabetes mellitus, chronic kidney disease, liver disease, as well as neurodegenerative and cardiovascular diseases. It has been reported that Cd exposure raises mortality by 17%. The health problems due to this metal could be explained by the induction of oxidative stress, inflammation, genomic instability, and endocrine system disruption (3). Cd causes cell damage through depleting the antioxidant content, such as glutathione and protein-bound sulfhydryl groups that results in the production of reactive oxygen species (ROS), including superoxide ions and hydroxyl radicals (4, 5). Initial studies have detected that Cd could induce irreversible detrimental effects on mammalian testis by disrupting the vascular system. Indeed, im-pacts on the seminiferous epithelium result in necrosis and testicular ischemia. It is assumed that the mam-malian testis is more sensitive than the other organs because of its unique vasculature (6).

For the production of efficient sperms by sperm-atogonia, the interplay between germ cells and differ-ent supporting somatic cells that make the spermato-genic niche is necessary. Among these cells, exclusi-vely Sertoli cells interact directly with germ cells and are vital for spermatogenesis. Consequently, the dysfunction of these cells often leads to spermatogenic failure (7). Sertoli cells with supportive and nourishing roles for germ cells in seminiferous tubules are involved in testis formation and spermatogenesis. Only a restricted number of germ cells could be supported by Sertoli cells and these cells are positively correlated with the daily sperm cell production (8). Various cells express different markers, which are usually specific to that cellular category. Some markers which are more expressed by Sertoli cells, namely Wilms' Tumor (WT1), Melan-A, CD99, and androgen receptor (AR), which are used for detecting Sertoli cells (9-11).

Testicular changes related to male infertility are a series of histopathological alterations in testis. There-fore, the present study focused on the possible spec-trum of these changes caused by Cd intoxication in rat testes and the possible role of Sertoli cells in processing the routes of Cd-induced testicular injury.

## Material and Methods

Cadmium chloride was purchased from Merck (Cat. no. 10108-64-2, USA). The CD99 antibody (Cat. No. IS057), Melan-A (Cat. No. M7196), and Wt1 antibody (Cat. No. IS055) were obtained from Dako Corporation, Carpentaria, California.

Animals

This study was approved by Kerman University of Medical Sciences Experimental Animals Local Ethics Committee and was carried out in the animal laboratory of this university. Sixteen adult male Wistar rats with the weight range of 220-240 g were purchased from the animal house of Afzalipour Kerman Medical School, Kerman, Iran, and were housed in the maintaining center at the room temperature (24-25℃) and humidity of about 50%-70% with the light/dark cycles of 12h/12h. Food and water were given ad libitum. About two weeks pre-experiment, the rats were acclimated to the laboratory. 

Experimental Design

The rats were randomly divided into two groups of control and Cd treatment with eight rats in each group. Cadmium chloride was administrated as a single dose of intraperitoneal injection (1.2 mg/kg BW). In the control group, normal saline was injected to rats. After 52 days, animals were euthanized with an intramuscular injection of ketamine hydrochloride (90 mg/kg BW) and xylazine hydrochloride (5 mg/kg BW). Next, the testicular tissues were excised and fixed in formalin for almost 6 h for histopathological evaluation. Next, these tissues were washed in phosphate-buffered saline (PBS) thrice for 5 min, cut into sections of 5-7 µm, and stained with Hematoxylin and Eosin. Images were captured with an upright light microscope. They were clarified based on the changes in seminiferous tubules, basement membrane thickness, spermatogenesis, and the presence or absence of Sertoli cells and Leydig cells. 

Immunohistochemistry (IHC)

Testis sections were stained with monoclonal antibodies targeting Wilms' Tumor (WT1), Melan-A, and CD99 by the avidin-biotin-peroxidase complex (ABC) kit. Following deparaffinization and rehydration, the sections were trypsinized (1 mg pronase/mL Tris-buffered saline) for 8 min and treated with 3% H_2_O_2_ for 30 min at room temperature followed by PBS washing three times. Afterwards, the sections were incubated with primary antibody for 60 min. The second antibody was added for 30 min followed by 30 min of incubation with ABC. Sections were washed completely with Tris-buffered saline (pH: 7.4) after each incubation. Counterstaining in hematoxylin was applied for sections using EnVision^TM^ FLEX Hematoxylin (Dako Autostainer/AutostainerPlus). The sections were visualized utilizing the EnVision FLEX, High pH (Dako Autostainer/Autostainer Plus).

## Results

Histopathological findings of the testes in the control group showed that all seminiferous tubules had a thin basement membrane and normal spermatogenic activity. The seminiferous tubules are composed of spermatogonia, round cells at the basement membrane which changed to primary and secondary spermatocytes, spermatids, and spermatozoa. Sertoli cells by elongated pyramid feature and vesicular nuclei were observed between other cells as supporting cells. Some Leydig cells were presented in the interstitial space of the testes. 

**Fig. 1:**
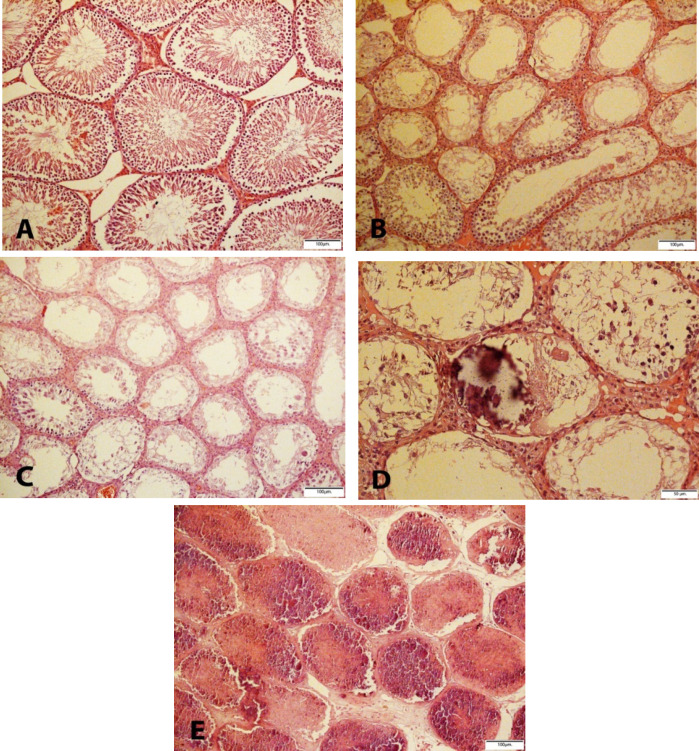
Testicular H & E staining A) Normal feature of seminiferous tubules in control group (10 X); B) Cadmium group: Spermatocytic arrest, germ cell aplasia and multinucleated giant Sertoli cell; C) Spermatocytic arrest, Germ cell aplasia, hypospermatogenesis, sloughing and disorganization and Sertoli cell pattern; D) Scattered Calcification and hyalinization of the seminiferous tubules, Scanty Sertoli cells and also Germ cells aplasia happened: E) Toxic infarction

**Fig. 2:**
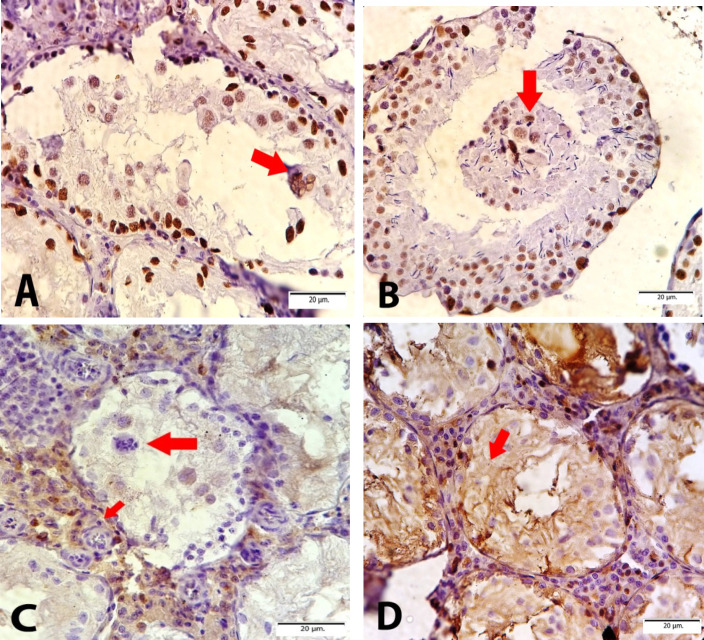
Immunohistochemistry staining, A & B) WT1 staining represents the Sertoli cells and multinucleated giant cells by browning their nuclei; C) Melan A staining show the Leydig cells in the interstitial space; D) Positive CD99 cytoplasmic staining of Sertoli cells

Cd-induced various damages in the testes with a variety of microscopic patterns, including sloughing and disorganization, incomplete spermatocytic arrest, spermatogenic hypoplasia, regional or incomplete fibrosis, tubular hyalinization, mixed atrophy, in addition to necrosis and calcification. Sloughing and disorganization mean that spermatogenesis has a disorderly appearance and the tubular lumina are filled with desquamated immature cells. Incomplete spermatocytic arrest is characterized by the arrest of spermatogenesis in some of the tubules. In the case of spermatogenic hypoplasia, tubules have a reduced population of germ cells and a poor order of spermatogenesis. In tubular hyalinization, the tubules have a small diameter and a markedly thickened basement membrane. Mixed atrophy is the synchronous occurrence of damaged seminiferous tubules containing germ cells and tubules with only Sertoli cells (Figure 1). Sometimes these mentioned changes were found as combinatory patterns and Leydig cells often had an increased number. In the tubules, few multinucleated giant cells of reactive nature were observed in the cases of testicular atrophy after Cd administration. The immunohistochemical (IHC) staining to find the multinucleated giant cells confirmed Sertoli cells and not spermatocytes (Figure 2). The WT1 and Melan-A staining revealed Sertoli cells in the basement membrane and lumina by a positive nuclear stain. The CD 99 was stained in the cytoplasm of Sertoli cells. 00000000000000

## Discussion

Based on the findings of Cd-induced spermatogenetic failure and review of the literature, we could confirm the range of changes from sloughing and disorganization to more severe changes, namely Sertoli cell-only and fibrosis patterns.

 The half-life of Cd is about 20-40 years in humans and accumulates in different human organs, such as kidney, liver, and testis during the time leading to disturbance. Augmentation has been described in exposure to lead as an environmental toxin. Exposure to low amounts of Cd alters the immunological micro-circumstances in the testis of laboratory animals, which enhances susceptibility to testicular autoimmunity. After almost three months of initial exposure to this pollutant, the testicular morphology and histopathology extremely changed with abnormal Leydig cells, atrophy of seminiferous tubules, fibrosis, and declined testicular size (12). 

In humans, testicular atrophy may result from a large variety of causes, including cryptorchidism, mumps orchitis (especially when the infection occurs at or after puberty), increased circulating endogenous estrogens not metabolized in liver cirrhosis, admini-stration of estrogens or gonadotropin-releasing hormone analogs for prostatic 

carcinoma, radiation exposure, chemotherapy, and exposure to various environmental toxins, such as Cd, arsenic, and lead (6, 12-15).

The results of the present study consistent with other experimental studies showed that a single dose of Cd could induce histopathological damages to the testis (12, 16). Histopathological findings entailed sloughing, disorganization, incomplete spermatocytic arrest, spermatogenic hypoplasia, regional or incomplete fibrosis, tubular hyalinization, atrophy, necrosis, and calcification. Moreover, multinucleated stromal giant cells were found by IHC staining and were confirmed as Sertoli cells. We can hypothesize that Sertoli cells, which originate from embryonic mesenchymal tissue, are the most resistant cells to Cd damge in the testicular tissue (17). Multinucleated stromal giant cells are a relatively common and clinically insignificant finding in the testes. They seem to be age-related and are said to be particularly common in testicular atrophy due to estrogen therapy (18).

Pathogenesis of Cd-induced toxicity causing spermatogenetic failure might be attributed to free radicals. Little evidence shows that Cd can affect the expression of distinct genes, such as transcription and translation factors, immediate early response genes, and stress-response genes. Cd can induce the gener-ation of ROS and reactive nitrogen species, which lead to toxic effects in many organs and tissues. Oxidative stress is one of the major mechanisms of Cd toxicity. By interfering with sulfhydryl groups, Cd influences not only the function of many different proteins, such as mitogen-activated protein kinases, but also the redox status of the cell. Therefore, the level of ROS elevates (19). It has been detected that malondialdehyde, as a product of lipid peroxidation, increased in Cd-exposed testis. The testis is more sensitive to peroxidative damages because of the high concentrations of poly-unsaturated fatty acids in the spermatozoa plasma membrane (19). 

The relationship between Cd and the parameters of semen quality has been proved in several studies. Cd causes damage to testes via altering molecular signaling pathways, such as p38, and increasing free radicals that disrupt the blood-testis barrier and antioxidant defense system, respectively (20). Egb-owon *et al.* in 2016 proposed that Cd ions enter Sertoli cells using Cd^2+/^Zn^2+^ transporters and activate the mitogen-activated protein kinase (MAPK) p38 pathway through the synthesis and release of cytokines. Furthermore, Cd damages cytoskeletal proteins and changes cell morphology and proliferation (21).

The effect of Cd concentration on semen quality seems to be controversial. Many investigations indicated a significant correlation between these two para-meters, while others did not (20, 22). Several rese-arches have shown that even at environmental concentration, Cd could diminish semen quality. This metal exerts deleterious impact by reducing semen quality and sperm parameters, such as motility, viability, sperm count, and morphology (23). In addition, Cd can impose toxicity through activating a number of signal transduction pathways, namely MAPK/phosphoinositide 3-kinase (PI3K)/c-Jun N-terminal kinase (c-JNK) signaling pathway that in turn disrupts blood-testis barrier and cell junction in the seminiferous epithelium (12).

Previous studies on animals have demonstrated that Cd decreased testicle weight, sperm count, and testostrone production (24-26). Cd reduces sperm count by perturbating the cell cycle, DNA repair system, and cell proliferation. Moreover, the deterioration of sperm count results from enhanced testosterone levels. It has been observed that even after the administration of a single dose of Cd, these negative changes could have happened (27). 

## Conclusion

Considering the importance of weakened male infertility and the impact of different concentrations of Cd on the testis, this study aimed to detect the impact of Cd injection on the histopathological changes of rat testes. We demonstrated a variety of changes from sloughing and disorganization to more severe changes, such as Sertoli cell-only and fibrosis pattern at the end of testicular damage. These findings could be matched with a different spectrum of histopathological changing in testis in male infertility workups.
